# Lung ultrasound guided management in chronic heart failure: an updated systematic review and meta-analysis of randomized controlled trials

**DOI:** 10.1093/ehjimp/qyag049

**Published:** 2026-03-18

**Authors:** Ravi Chotalia, Kevin Mohee, Robert Ambrogetti, Minesh Chotalia, Latif Raiyan Rahman, Hasan Mohiaddin

**Affiliations:** Lincoln Heart Centre, United Lincolnshire Hospitals NHS Trust, Greetwell Road, Lincoln, LN2 5QY, UK; University of Lincoln, Brayford Pool, Lincoln, LN6 7TS, UK; Cardiology Department, Glenfield Hospital, Groby Road, Leicester, UK; Cardiology Department, Wycombe Hospital, Wycombe, UK; Birmingham Acute Care Research Group, University of Birmingham, Birmingham, UK; Department of Anaesthetics and Critical Care, Queen Elizabeth Hospital, Mindelsohn way, Birmingham, B15 2GW, UK; Acute Medicine Department, University Hospitals of Leicester, Leicester, UK; Cardiology Department, Glenfield Hospital, Groby Road, Leicester, UK; NIHR Leicester Biomedical Research Centre and Department of Cardiovascular Sciences, University of Leicester, Leicester, UK

**Keywords:** lung ultrasound, chronic heart failure, heart failure hospitalizations, heart failure urgent visits

## Abstract

**Introduction:**

Existing systematic reviews support the prognostic and therapeutic value of lung ultrasound (LUS) in heart failure (HF), but recent randomized controlled trials (RCTs) in chronic HF justify an up-to-date synthesis.

**Methods and results:**

A systematic search of OVID via Medline, SCOPUS, COCHRANE, and CINAHL was conducted from inception until 25 February 2025. The study was registered with PROSPERO (ID: CRD420251003434). RCTs of LUS interventions in patients with chronic HF were included. The primary outcomes were HF urgent visits and HF hospitalizations. Secondary outcomes included mortality, hypokalaemia, and worsening renal function. Five RCTs, involving a total of 694 patients, were included in meta-analyses, with variability in LUS-based definitions of pulmonary congestion across studies. LUS-guided management was associated with a significant reduction in HF urgent visits [RR 0.31 (95% CI 0.17, 0.55), I^2^ = 0%] and a nonsignificant improvement in HF hospitalizations [RR 0.76 (95% CI 0.48, 1.18), I^2^ = 38.9%]. There was no difference in the rates of mortality, hypokalaemia, or worsening renal function.

**Conclusion:**

LUS-guided management is associated with a significant reduction in urgent HF visits and a nonsignificant reduction in HF hospitalizations, with no difference in rates of mortality, hypokalaemia, or worsening renal function. Future studies should aim to establish an optimal, standardized LUS-based definition of pulmonary congestion in chronic HF.

## Graphical Abstract

**Graphical Abstract:**
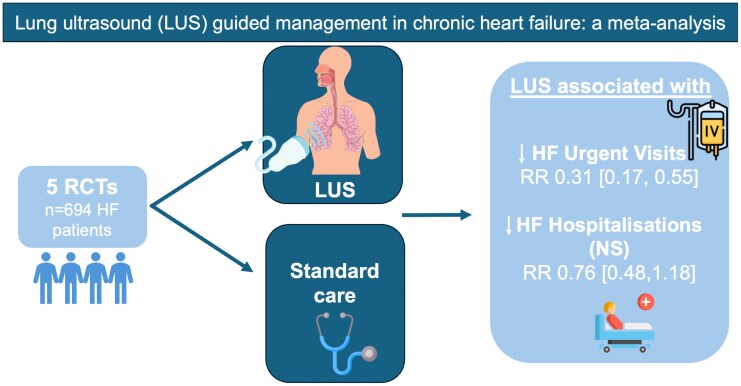
Lung ultrasound (LUS) in chronic heart failure (HF). This systematic review and meta-analysis assessed randomized controlled trials (RCTs) of LUS vs. standard care in the treatment of chronic HF. NS: nonsignificant; RR: risk ratio, 95% confidence intervals displayed.

## Introduction

Heart failure (HF) is a common and progressive condition that affects approximately 64 million people worldwide.^1^ The number of patients with HF is increasing due to several factors, including improvements in diagnosis, an ageing population and improved survival after diagnosis.^2^

Decompensated HF, resulting in HF hospitalizations or urgent HF visits, is the rapid onset or change in symptoms or signs of HF. HF hospitalizations continue to rise in incidence and have become the leading cause of hospitalization in patients over 65 years of age.^3^ Associated with significant morbidity and mortality, it also carries a huge financial burden, with over 80% of the cost of HF originating from hospitalizations or urgent HF visits.^4^ As a result, strategies to reduce HF hospitalizations and urgent HF visits are of considerable research interest.

**Figure 1:**
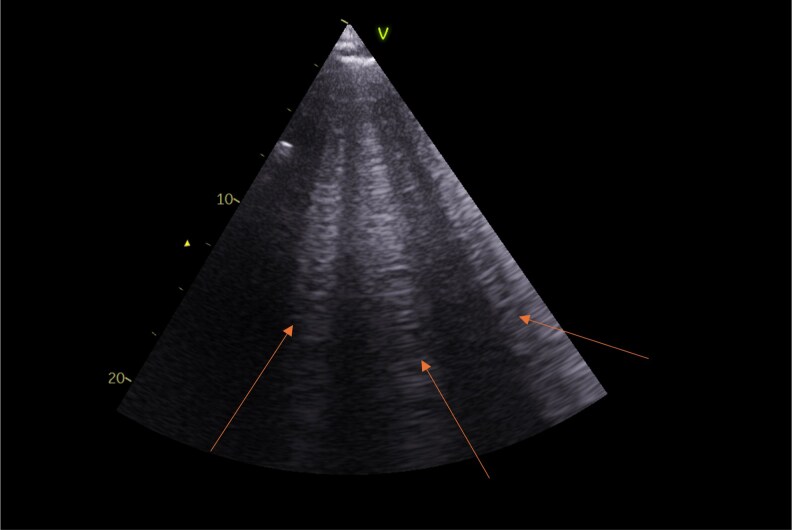
Lung ultrasound depicting B-lines (indicated by red arrows). A total of three B-lines are visible. Appropriate written consent was obtained from the patient for acquisition and use of this image.

Pulmonary congestion is a characteristic feature of HF decompensation. However, traditional physical examination, such as auscultation for crackles or assessment of jugular venous pressure, has a limited sensitivity in identifying signs of decompensated HF.^5^ Point of care lung ultrasound (LUS) is a diagnostic tool that can be used to identify pulmonary congestion in HF. LUS detects B-lines (*Figure 1*), which are vertical reverberation artefacts that correlate with extravascular lung water, and has shown a high sensitivity in detecting pulmonary congestion.^6^ Moreover, multiple randomized controlled trials (RCTs) have identified that the use of LUS can improve outcomes in acute^7^ and chronic HF.^7,8^ This has led to the recognition of the benefits of LUS by the European Society of Cardiovascular Imaging in a 2023 consensus statement.^9^ However, the most up-to-date European Society of Cardiology HF guidelines^10,11^ do not provide a recommendation for its use in chronic HF, but note it may be considered in the setting of acute HF presentations. LUS may also be incorporated within a multiorgan ultrasound assessment of congestion, recognizing that pulmonary congestion represents only one component of the systemic congestive phenotype in HF, as demonstrated by Pugiliese *et al.*^12^

A prior meta-analysis assessed the use of LUS in chronic HF^8^ and found a nonsignificant trend toward reduced HF hospitalizations and a significant reduction in urgent HF visits. Since that publication, further RCTs^13,14^ have been published. Therefore, we conducted an updated meta-analysis of LUS-guided management in patients with chronic HF, providing an up-to-date synthesis of its impact on HF related clinical outcomes.

## Methods

This systematic review was conducted by adhering to the criteria defined in the Cochrane Handbook for Systematic Reviews of Interventions^15^ and reported using the Preferred Reporting Items for Systematic Reviews and Meta-Analysis (PRISMA) 2020 statement.^16^ The protocol was registered with PROSPERO under the ID: CRD420251003434.

### Search strategy

A systematic search was conducted from inception to 25 February 2025 using the following databases: OVID via Medline, SCOPUS, COCHRANE, and CINAHL. The full details of the search strategy and MeSH terms are included as Supplementary Material  *S1*. Reference lists of other systematic reviews and RCTs were also screened.

### Eligibility

We only included RCTs that compared the efficacy of LUS-guided outpatient management and usual care in adults ≥18 years old with HF. Only trials that reported the primary and/or secondary outcomes of interest were included in the review.

Duplicate records were automatically identified by Rayyan, followed by manual review and removal by the reviewers. Titles and abstracts were independently screened by two reviewers (R.A. and H.M.), with any conflicts resolved by discussion. All nonrandomized studies and articles not written in English were excluded.

### Data extraction

Data were extracted from selected studies by two independent reviewers (H.M. and R.C.). Data, including study characteristics (author, year, and country), patient characteristics (sample size, demographic data, HF aetiology), type of LUS intervention (length of intervention, length of follow up, definition of congestion), and reported outcome measures (HF urgent visit rates, HF hospitalization rates, mortality rates and other secondary outcomes) were collected.

### Primary outcome

The primary outcomes were HF-related urgent visits (defined as a same day or ambulatory urgent appointment that did not meet the threshold for hospital admission) and HF-related hospitalization. These outcomes were analysed and reported separately. Risk ratios were calculated with 95% confidence intervals (CIs), comparing the LUS-guided management with usual care.

### Secondary outcomes

Secondary outcomes included mortality, hypokalaemia, and worsening renal function. These outcomes were compared between the LUS- guided cohort and usual care cohort and expressed as risk ratios with 95% CIs. Studies that did not report data on the secondary outcome of interest were excluded from the corresponding analysis.

### Bias assessment

The risk of bias assessment was conducted independently by two reviewers (R.C. and M.C.), using the Cochrane risk of bias 2 tool.^17^ Any conflicts were resolved by discussion with a third reviewer who was not involved in the initial assessment. The risk of bias plots was generated using the robvis tool.^18^

### Statistical analysis

Meta-analyses were performed for each outcome of interest using the random-effects model. Statistical heterogeneity was assessed using the *I*^2^ statistic, with an *I*^2^ of 0–25% considered low, 26–50% moderate, and >50% considered high heterogeneity. Meta-regression could not be performed due to the small number of studies within the meta-analysis. All analyses were performed using Stata18 (StataCorp, College Station, TX).

## Results

**Figure 2:**
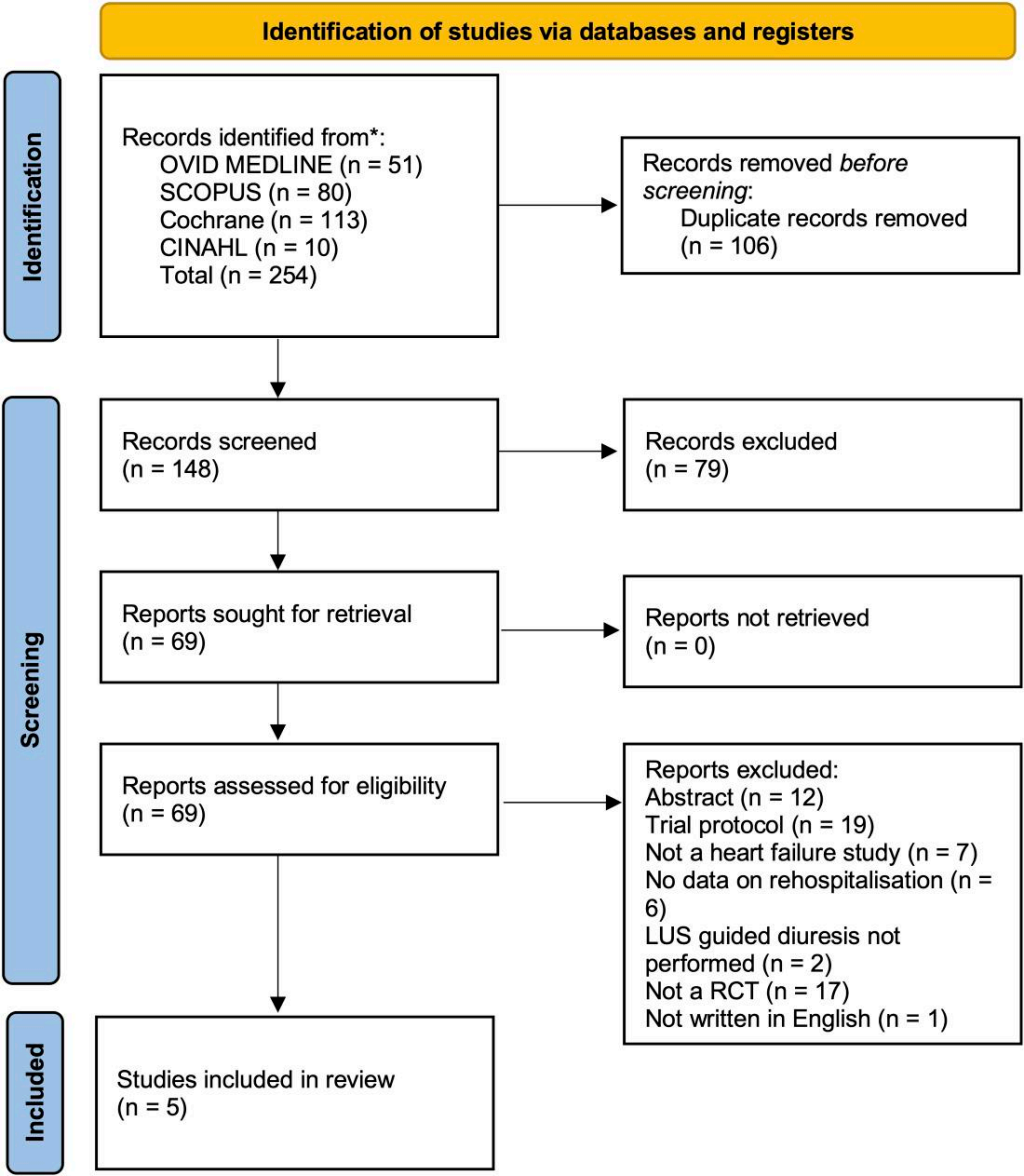
Prisma flow diagram of study selection. Flow diagram showing the identification, screening, eligibility, and inclusion of studies for the systematic review.

After removing duplicate articles (*n* = 106) and following title and abstract screening, a total of 69 records were identified for full-text review (*Figure 2*). Of these records, 64 were excluded for the reasons listed in *Figure 2*. The remaining 5 studies^13,14,19–21^ were included in the systematic review and meta-analysis, involving 694 HF patients.

*Table 1* shows a detailed description of the five included studies published between 2019 and 24. The median age of patients ranged from 62 to 80 years, and the prevalence of male participants ranged from 45% to 81% (*Table 1*). Two of the included studies originated in Spain^14,19^ and one study was from Italy,^20^ Mexico,^21^ and Australia.^13^

**Table 1:** Studies examining the use of lung ultrasound in outpatient management of HF patients and their key characteristics and outcomes

Study	LUS-HF^19^	CLUSTER-HF^21^	Marini *et al*.^20^	EPICC^14^	Zisis *et al*.^13^
Year of publication	2019	2020	2020	2022	2024
Country	Spain	Mexico	Italy	Spain	Australia
HF population	Patients hospitalized with HF	Patients hospitalized with HF	Chronic HF on OMT EF <45%	Patients hospitalized with HF	Patients hospitalized with HF
Follow-up (months)	6	6	3	6	3
Definition of congestion	Total number of B-lines across 8 chest zones ≥3	Total number of B-lines across 8 chest zones ≥3	Lung comet score definition^20^	Bilateral presence of B-lines in one pulmonary region and/or significant pleural effusion (>1 cm)	Total number of B-lines across 8 chest zones ≥10
LUS group treatment regimen	Follow-up at 14 days, 1 month, 3 months and 6 months.	Follow-up at day 14, 6 weeks and 3 and 6 months.	Follow-up not specified	Follow up at week 1–2, and 1,3 and 6 months. ^a^	Follow up at week 1–2, week 3 and 1 month
Control group treatment regimen	Follow-up at 14 days, 1 month, 3 months and 6 months	Follow-up at day 14, 6 weeks and 3 and 6 months	Follow-up not specified	Follow-up at week 1–2, and 1,3 and 6 months^a^	Follow-up at week 4 with phone-call assessment
Primary outcome	Composite of urgent HF visits, HF hospitalization or all-cause death	Composite of urgent HF visits, HF hospitalization or all-cause death	HF hospitalization	Composite of urgent HF visit, HF hospitalization, CV death	Composite of HF hospitalization or all-cause death
Primary outcome result	Significant reduction in LUS group	Significant reduction in LUS group	Significant reduction in LUS group	No significant difference between groups	No significant difference between groups
Secondary outcome result	Significant reduction in urgent HF visits in LUS group. No significant difference in HF hospitalizations or death. No difference in rates of hypokalaemia or worsening renal function. Significant improvement in 6MWD and QoL scores in LUS group	Significant difference in urgent HF visits in LUS group. No significant difference in HF hospitalizations or death. No difference in rates of hypokalaemia or worsening renal function or QoL scores	No significant difference in mortality. Significant difference in NT-proBNP value and QoL scores in LUS group	No significant difference in HF hospitalization, ED visits, all-cause mortality, worsening renal function, or QoL scores	No significant difference in HF hospitalization or death.

^a^Optional further visits could be organized as per clinician.

OMT: optimal medical therapy, 6MWD: 6 min walk test distance, QoL: quality-of-life, EF: ejection fraction.

All studies examined the use of LUS vs. usual care in the outpatient management of HF patients. All of the studies except Marini *et al.*^20^ examined LUS in all HF patients recently hospitalized for decompensated HF. In contrast, Marini *et al.* studied chronic patients without recent hospitalization, but who only had HF with reduced ejection fraction. Usual care varied across studies: in three studies, patients had equivalent follow-up to the LUS group^14,19,21^ and had LUS performed, but the results were not available to clinicians. The usual care group in Zisis *et al.*^13^ had a reduced follow-up intensity compared to the LUS group (only at week 4 with a telephone assessment). Marini *et al*.^20^ did not report the frequency or nature of follow up appointments in either LUS-guided or usual care groups. Length of follow up varied, with three studies following up to 6 months^14,19,21^ and two studies following up to 3 months.^13,20^ The definition of congestion varied across studies (see *Table 1*).

Regarding outcome measures, the primary outcome of three studies was a composite of urgent HF visits, HF hospitalizations, or all-cause death.^14,19,21^ The primary outcome in Zisis *et al*. was a composite of HF hospitalizations or CV death,^13^ whereas the primary outcome in Marini *et al*. was HF hospitalizations.^20^ All studies reported HF hospitalizations and mortality. Three studies reported urgent HF visits separately.^14,19,21^ Difference in quality of life was reported in four studies;^14,19–21^ however, these results could not be synthesized as different quality of life tools were used.

### Quality assessment

**Figure 3:**
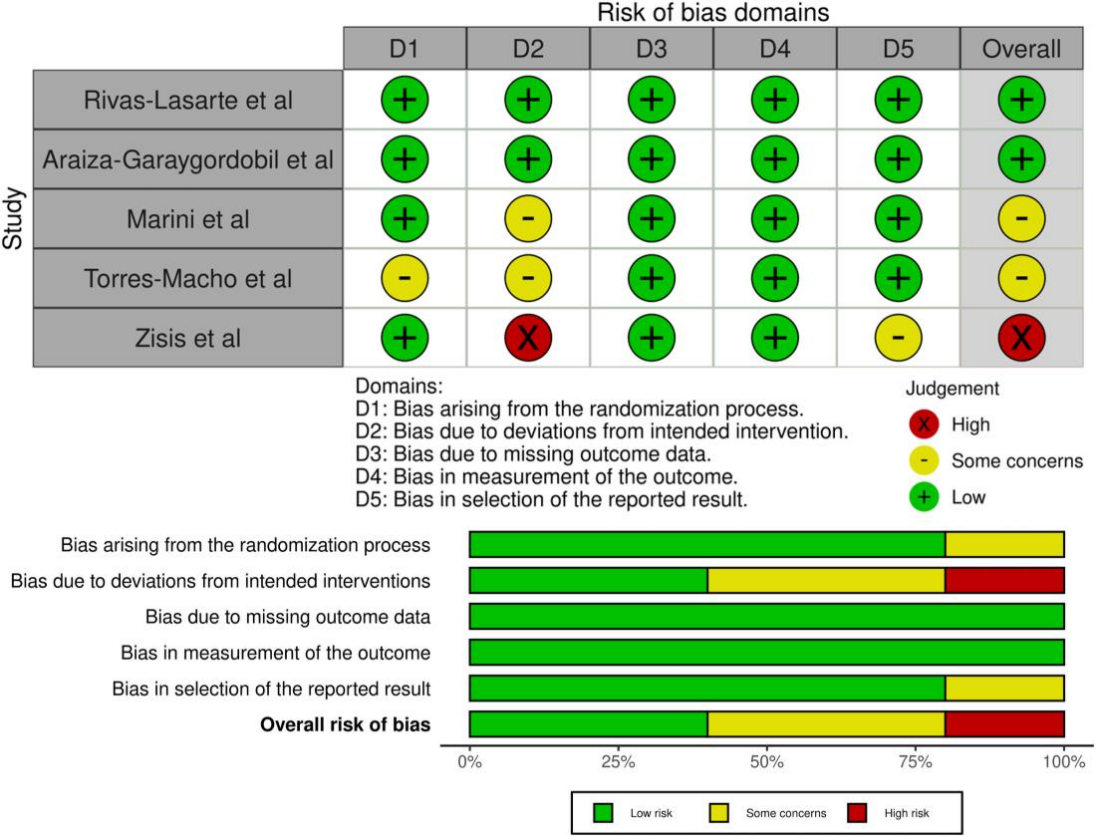
Risk of bias assessment of the included studies using ROB 2.0 tool. Risk of bias plots were generated using the robvis tool.

Two studies were deemed to be low risk of bias,^19,21^ two were judged to have some concerns,^14,20^ and one was deemed to be high risk.^13^ Frequent sources of bias originated from domain 2 (bias originating from deviations from intended interventions), due to patients being unblinded, and extra follow-up for the LUS cohort compared to usual care, which could have influenced results. *Figure 3* illustrates a detailed risk of bias assessment.

### Primary outcomes

**Figure 4:**
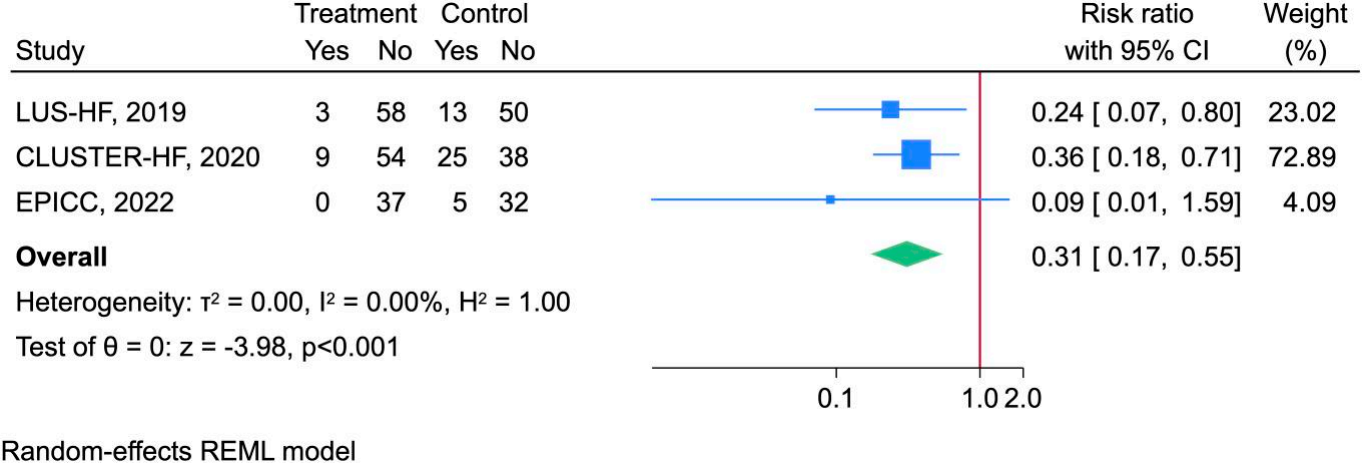
Forest plot illustrating the effect of LUS interventions compared to usual care on HF urgent visits during follow-up period.

A meta-analysis of three studies^14,19,21^ showed a significant reduction in proportion of patients with urgent HF visits [RR 0.31 (95% CI 0.17, 0.55), *I*^2^ = 0%] (*Figure 4*).

**Figure 5:**
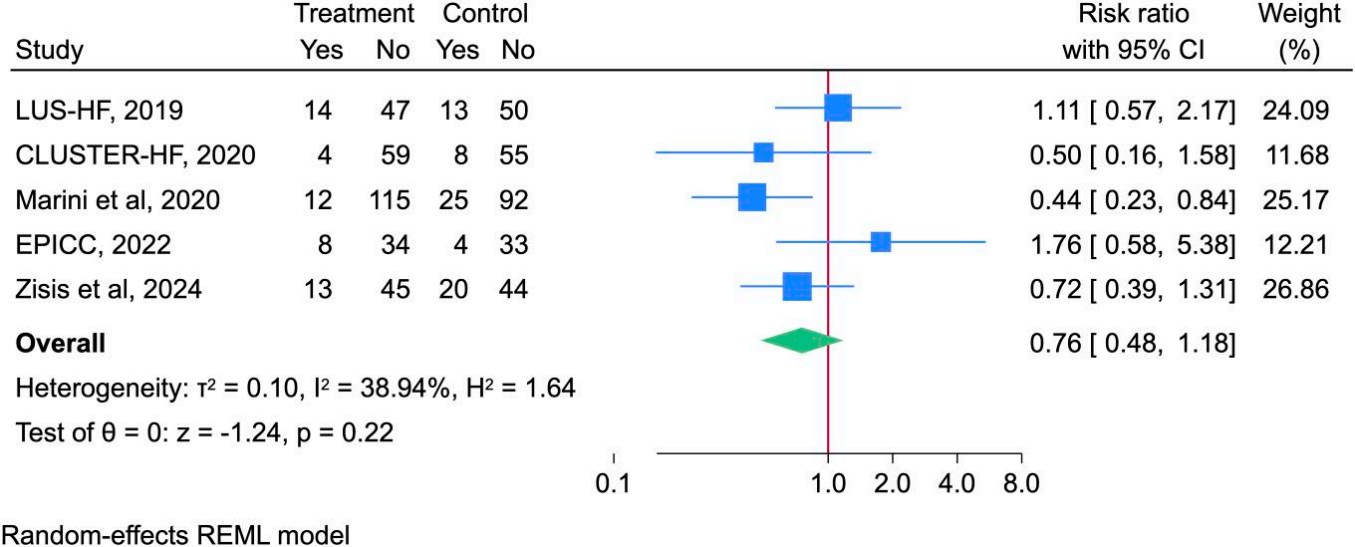
Forest plot illustrating the effect of LUS interventions compared to usual care on HF hospitalizations during follow-up period.

A meta-analysis of five studies showed that whilst there was a trend towards reduced HF hospitalizations with LUS-guided management, this was not significant [RR 0.76 (95% CI 0.48, 1.18), *I*^2^ = 38.9%] (*Figure 5*).

### Secondary outcomes

There was no significant difference in rates of mortality with LUS use [RR 1.25 (95% CI 0.73, 2.14), *I^2^* = 0%] (see Supplementary data online, *Figure S1*). The number of patients with hypokalaemia was reported in two studies.^19,21^ A meta-analysis of these two studies showed no significant difference in rates of hypokalaemia with LUS use [RR 0.72 (95% CI 0.21, 2.42), I^2^ = 39.4%] (see Supplementary data online, *Figure S2*). The number of patients with worsening renal function was reported in three studies.^14,19,21^ A meta-analysis of these  three studies showed no significant difference in rates of worsening renal function with LUS use [RR 1.21 (95% CI 0.69, 2.15), I^2^ = 0%] (see Supplementary data online, *Figure S3*).

## Discussion

In this meta-analysis, we examined the impact of LUS-guided management in patients with chronic HF. Our findings indicate that the use of LUS is associated with a significant reduction in urgent HF visits compared with usual care, alongside a nonsignificant reduction in HF hospitalizations. This effect is most likely due to the ability of LUS to detect subclinical pulmonary congestion not apparent on clinical examination, allowing clinicians to more appropriately titrate diuretic therapy. LUS-guided therapy in HF appears safe, with no significant difference between groups in secondary outcomes, including mortality, hypokalaemia, or worsening renal function.

The previous meta-analysis by Mhanna *et al.*^8^ included three RCTs and found LUS-guided management significantly reduced urgent HF visits [RR 0.32 (95% CI 0.18–0.59), *P* = 0.0002] and, although there was a trend to reduction in HF hospitalizations, this was not significant [RR 0.65 (95% CI 0.34–1.22), *P* = 0.18]. Our analysis extends these findings by incorporating additional data from Torres-Macho *et al.*^14^ and Zisis *et al.*^13^

The effects of LUS-guided management on HF hospitalizations and urgent visits varied across studies, with some trials demonstrating statistically significant reductions and others showing nonsignificant trends. Several factors may account for this variability. First, studies varied in how pulmonary congestion was defined on LUS. Zisis *et al.*^13^ used threshold of >10 B-lines across eight lung zones to define congestion, whereas Torres-Macho *et al.*^14^ required bilateral positive lung zones, with each zone having ≥3 B-lines. LUS-HF and CLUSTER-HF^19,21^ used a threshold of ≥3 B-lines in total across all lung zones, and Marini *et al.* applied the lung comet score.^20^ Differences in these thresholds likely affected which patients were identified for intervention and may have influenced the magnitude of observed benefit. Standardizing B-line thresholds in future studies may improve comparability.

Second, patient characteristics varied. Studies such as Torres-Macho *et al.* and Zisis *et al.* included older patients (81 and 76 years, respectively) and higher proportion of patients with CKD (48% and 57%, respectively) compared with prior studies (average age 62.5–71.5 years; CKD 27–36%). These factors can reduce responsiveness to diuretics^22^ and may blunt the impact of LUS-guided therapy.

Third, trial size and recruitment challenges, including those caused by the COVID-19 pandemic, may have limited statistical power. For example, Torres-Macho^14^ enrolled 79 of 152 planned patients, and Zisis *et al*.^13^ enrolled 122 of the 404 planned patients, which may have increased the risk of type 2 error and contribute to nonsignificant findings in some outcomes.

### Strengths and limitations

There are multiple strengths of our study. Our search was conducted across multiple major databases. Moreover, the study protocol was prespecified and registered, reducing risk of bias in study selection and analysis. Only RCTs were included, which represent the highest level of clinical evidence. Most outcomes demonstrated low heterogeneity, e.g. *I*^2^ = 0% for urgent HF visits, mortality, and worsening renal function. In contrast, moderate heterogeneity was observed for HF hospitalizations, *I*^2^ = 38.9%, indicating some between study variability. While low heterogeneity suggests consistency of effect estimates due to broadly similar study designs and outcome definitions across studies, the absence of detectable heterogeneity is uncommon in clinical meta-analyses and should be interpreted cautiously, as it could reflect a limited statistical power to detect heterogeneity due to the small number of included studies. Further limitations include the inclusion of two trials that were underpowered, and one study was judged to be at high risk of bias, which may limit the precision and robustness of our results.

### Clinical implications

Our results suggest that the use of LUS can be effective in reducing HF urgent visits and possibly hospitalizations. Most included studies recruited HF patients after an acute decompensation; however, Marini *et al.* also assessed the use of LUS in chronic HF outpatients with a reduced ejection fraction. From a health system perspective, LUS- guided care is rapid, relatively inexpensive, and a simple technique that can be taught to nonspecialists to perform with good accuracy following brief, structured training.^23,24^ This evidence supports LUS as a safe, scalable adjunct in outpatient HF care.

### Future research

Further studies are required to assess which patient populations derive the greatest benefit from the use of LUS. The results from these trials suggest the use of LUS is equally beneficial across different HF ejection fractions and in chronic outpatients vs. postdischarge populations. However, its benefit may be reduced in older patients and in those with CKD, highlighting the value of dedicated trials in older, comorbid populations. It is also important to note that pulmonary congestion is defined inconsistently across LUS trials. Establishing a standardized definition, and identifying which most accurately reflects congestion, is essential. It is also increasingly recognized that pulmonary congestion may only be one aspect of a multiorgan process of decompensation in HF.^12,25^ Indeed, ultrasound assessment of systemic venous congestion, such as inferior vena cava size and renal venous flow patterns, have been shown to carry independent and incremental prognostic information in HF, in addition to LUS assessment.^12,25^ It is possible that integrating ultrasound assessment of pulmonary congestion and systemic venous congestion into a combined assessment may help to better identify patients at risk of decompensation and guide therapy, although this requires further study.

## Conclusion

In conclusion, our meta-analysis demonstrated that outpatient LUS-guided management is associated with a significant reduction in urgent HF visits and a nonsignificant reduction in HF hospitalizations. Importantly, LUS-guided management appears safe, with no observed increases in mortality, hypokalaemia, or worsening renal function. Future studies should focus on establishing an optimal, standardized LUS-based definition of pulmonary congestion in chronic HF to improve comparability and clinical application.

## Supplementary Material

qyag049_Supplementary_Data

## Data Availability

This meta-analysis used data extracted from previously published studies, which are cited in the reference list. All data generated through the analysis, including pooled estimates, summary tables, and forest plots, are included in the article and its supplementary materials. The dataset of extracted study-level data can be made available from the corresponding author upon reasonable request.

## Lead author biography

Dr Ravi Chotalia is an early career clinician–researcher with an interest in heart failure and interventions aimed at reducing heart failure admissions. His other research interests include fractional flow reserve derived from CT and how it can predict adverse outcomes. When not at the hospital or working on research, Ravi is either spending time with his wife or at the gym.
