# Supplementary material for: Lung ultrasound guided management in chronic heart failure: an updated systematic review and meta-analysis of randomized controlled trials
Source: Eur Heart J Imaging Methods Pract. 2026 Mar 18;4(1):qyag049. doi: 10.1093/ehjimp/qyag049 (PMC13007594; doi:10.1093/ehjimp/qyag049)
Supplement: qyag049_Supplementary_Data [file qyag049_supplementary_data.zip › qyag049_Supplementary_figure_lgdends.docx]

Supplementary figure 1: Forest plot illustrating the effect of LUS interventions compared to usual care on mortality during follow up period

Supplementary figure 2: Forest plot illustrating the effect of LUS interventions compared to usual care on hypokalaemia during follow up period

Supplementary figure 3: Forest plot illustrating the effect of LUS interventions compared to usual care on worsening renal function during follow up period
