# Supplementary material for: Lung ultrasound guided management in chronic heart failure: an updated systematic review and meta-analysis of randomized controlled trials
Source: Eur Heart J Imaging Methods Pract. 2026 Mar 18;4(1):qyag049. doi: 10.1093/ehjimp/qyag049 (PMC13007594; doi:10.1093/ehjimp/qyag049)
Supplement: qyag049_Supplementary_Data [file qyag049_supplementary_data.zip › Search terms- supplementary materal 1.docx]

The following search terms were used: ( "lung ultrasound" or "thoracic ultrasound" ), ( "pulmonary edema" or "pulmonary oedema" or "heart failure" ), ( randomised or randomized or RCT ).
