# Supplementary figures and images for: Lung ultrasound guided management in chronic heart failure: an updated systematic review and meta-analysis of randomized controlled trials
Source: Eur Heart J Imaging Methods Pract. 2026 Mar 18;4(1):qyag049. doi: 10.1093/ehjimp/qyag049 (PMC13007594; doi:10.1093/ehjimp/qyag049)

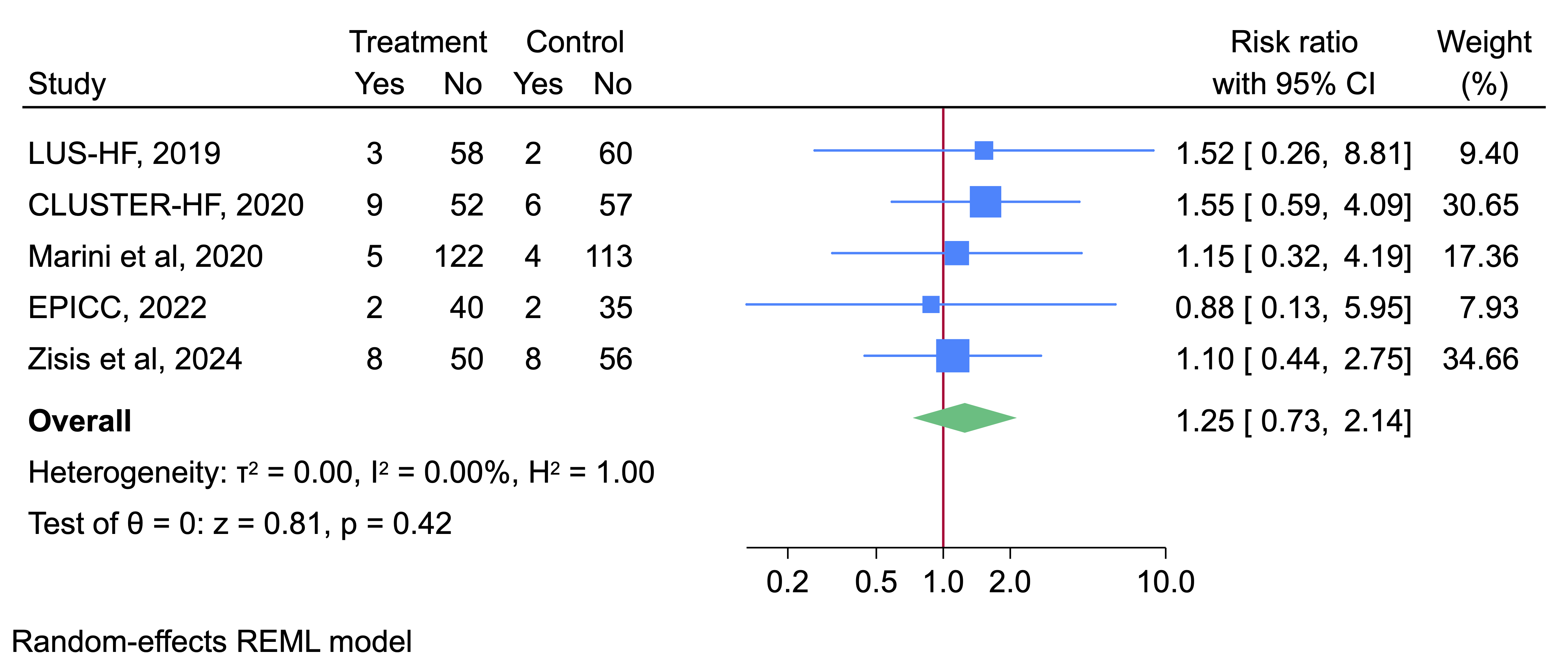

Supplement: qyag049_Supplementary_Data [file qyag049_supplementary_data.zip › supplementary figure 1.tiff]

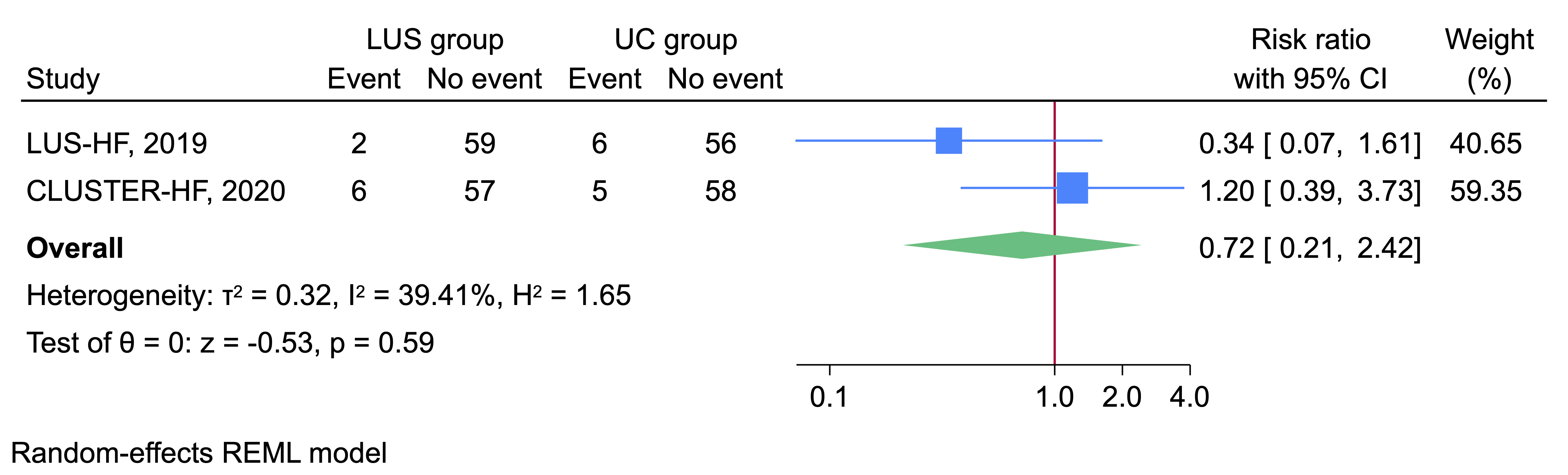

Supplement: qyag049_Supplementary_Data [file qyag049_supplementary_data.zip › Supplementary Figure 2.tiff]

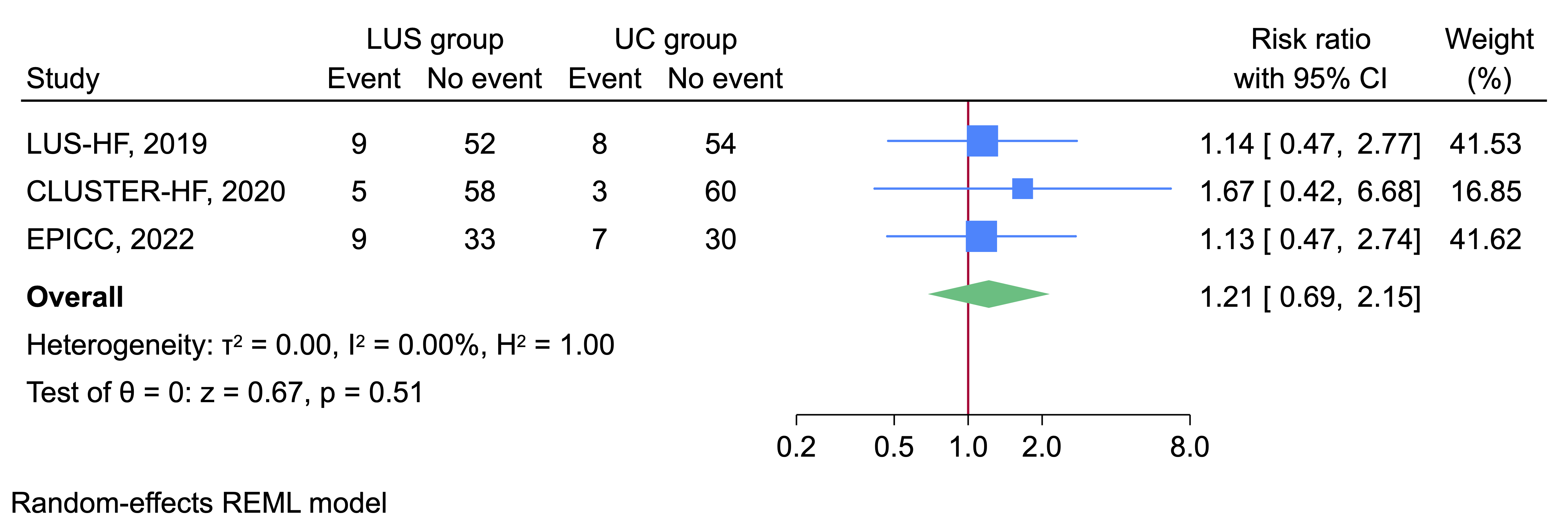

Supplement: qyag049_Supplementary_Data [file qyag049_supplementary_data.zip › Supplementary Figure 3.tiff]
